# Prognosis in Primary Amenorrhœa

**Published:** 1901-02-16

**Authors:** 


### PROGNOSIS IN PRIMARY AMENORRHŒA.

FROM a careful study of 1,000 cases in which he had noted the age at which menstruation started, Dr. Arthur E. Giles[1] has worked out various interesting statistics bearing on the question of prognosis as to the establishment of menstruation in cases of primary amenorrhœa, or delayed menstruation, and as to the likelihood of pregnancy occurring in such cases. Cases of concealed menstruation from obstruction in the passages are, of course, put on one side. He shows that of his 1,000 cases the following were the relative frequency of ages for the onset of menstruation:—

| | Cases. | |
|---|---|---|
| At 8 years | 0 | = 5·5 per cent. |
| „ 9 „ | 5 | |
| „ 10 „ | 13 | |
| „ 11 „ | 37 | |
| „ 12 „ | 85 | = 88·4 per cent. |
| „ 13 „ | 188 | |
| „ 14 „ | 211 | |
| „ 15 „ | 173 | |
| „ 16 „ | 151 | |
| „ 17 „ | 76 | |
| „ 18 „ | 28 | = 6·1 per cent. |
| „ 19 „ | 18 | |
| „ 20 „ | 6 | |
| „ 21 „ | 3 | |
| „ 22 „ | 3 | |
| „ 23 „ | 1 | |
| „ 24 „ | 0 | |
| „ 25 „ | 1 | |
| „ 26 „ | 0 | |
| „ 27 „ | 1 | |

Thus we see that 93·5 per cent. of the cases begin to menstruate before 18 years of age, and we may consider all cases in which menstruation does not begin till the age of 18 or later as cases of "late menstruation."

The next question then is, What is the prognosis as to menstruation and as to the possibility of child-bearing in cases of late menstruation? From a consideration of the details given as to the individual cases forming this group it may be stated that the prevailing character of late menstruation is that it is scanty, irregular, and painless; but this does not mean that cases do not occur in which the character is exactly the opposite of that which generally prevails. Bearing in mind the table given above we may say that even without examination we can give a good prognosis so far as the establishment of menstruation is concerned in the case of a girl of 16 or 17; at 18 or 19 the prognosis must be more guarded and the effect of treatment must be watched; while after the age of 20 no opinion should be expressed without making an examination.

Then we must be guided to some extent by the general health of the patient. If she be suffering from anæmia, tuberculosis, or some other constitutional condition that may cause amenorrhœa we may hope (with the proviso of age just considered) that menstruation will begin when the general condition has improved.

But supposing the general condition to be good, and the patient 20 years of age, or older, the development of breasts and pubic hair should be noted (for any marked deficiency in these secondary sexual characters is unfavourable), and a pelvic examination should be made—under an anæsthetic if unmarried. If no abnormality can be found, or the uterus be only slightly under the normal size, the prognosis is not unfavourable; on the other hand if the uterus is very small, or if the ovaries are very small and infantile in shape, the probability is against the establishment of menstruation.

With regard to the child-bearing, if the pelvic organs are found to be normal we can say that if the patient be 18 and menstruation has not yet appeared the prognosis is still fairly good; if she is 19, she is more likely to be relatively, although not absolutely, unfertile; while if 20 or over the likelihood of child bearing rapidly diminishes in proportion to age. If she is 25, and still has not menstruated, she will almost certainly be sterile, as also will be the case if the uterus is undeveloped, the uterine canal measuring 2 inches or less.

[1] Clin. Jour., Jan. 30.

